# The genome sequence of the Madeiran Speckled Wood, *Pararge xiphia* Hübner, 1819 (Lepidoptera: Nymphalidae)

**DOI:** 10.12688/wellcomeopenres.25814.1

**Published:** 2026-02-09

**Authors:** Valéria Marques, Roger Vila, Charlotte J. Wright, Joana I. Meier, Mark L. Blaxter, Wellcome Sanger Institute Tree of Life Management, Samples and Laboratory team, Wellcome Sanger Institute Scientific Operations: Sequencing Operations, Wellcome Sanger Institute Tree of Life Core Informatics team, Tree of Life Core Informatics collective, Project Psyche Community

**Affiliations:** 1 Institut de Biologia Evolutiva (CSIC-UPF), Barcelone, Catalonia, Spain; 2 Tree of Life Programme, Wellcome Sanger Institute, Hinxton, England, UK

**Keywords:** Pararge xiphia; Madeiran Speckled Wood; genome sequence; chromosomal; Lepidoptera

## Abstract

We present a genome assembly from a male specimen of *Pararge xiphia* (Madeiran Speckled Wood; Arthropoda; Insecta; Lepidoptera; Nymphalidae). The assembly contains two haplotypes with total lengths of 528.41 megabases and 523.51 megabases. Most of haplotype 1 (99.02%) is scaffolded into 28 chromosomal pseudomolecules, including the Z sex chromosome. Haplotype 2 was assembled to scaffold level. The mitochondrial genome has also been assembled, with a length of 15.27 kilobases. This work is part of Project Psyche, a collaborative programme generating genomes for European butterflies and moths.

## Species taxonomy

Eukaryota; Opisthokonta; Metazoa; Eumetazoa; Bilateria; Protostomia; Ecdysozoa; Panarthropoda; Arthropoda; Mandibulata; Pancrustacea; Hexapoda; Insecta; Dicondylia; Pterygota; Neoptera; Endopterygota; Amphiesmenoptera; Lepidoptera; Glossata; Neolepidoptera; Heteroneura; Ditrysia; Obtectomera; Papilionoidea; Nymphalidae; Satyrinae; Satyrini; Parargina; *Pararge*; *Pararge xiphia* Hübner, 1819 (NCBI:txid111933)

## Background

The Madeiran Speckled Wood, *Pararge xiphia*, is a nymphalid butterfly endemic to the island of Madeira. It is a medium to large butterfly, dark brown with orange spots. It may be confused with its European congener, the Speckled Wood, *Pararge aegeria*; the main differences are a slightly more convex, mostly brown outer margin of the forewing, a small but distinctive white mark on the marbled brown underside, and two eyespots on the upperside of the hindwing.

*P. xiphia* is on the wing all year round and can be found from sea level up to 1 000 m a.s.l. within clearings and around the edges of the native laurel and chestnut forest. The larvae feed on several grass species in the family Gramineae, including *Agrostis gigantea*, *Brachypodium sylvaticum*, *Holcus lanatus* and the endemic *Festuca donax* ( Aguiar & Karsholt, 2006), and live on the broad grass leaves, constructing a seat web.

*P. xiphia* is listed as Near Threatened in the IUCN Red List ( van Swaay *et al.*, 2025). It is endangered by housing sprawl, intensive agriculture with large fields and fewer structures, forestry (plantation of non-native tree species), and competition with *P. aegeria*, which was introduced to the island in 1976 and is seemingly more resistant to alien tree species ( Jones *et al.*, 1998).

**Figure 1.**
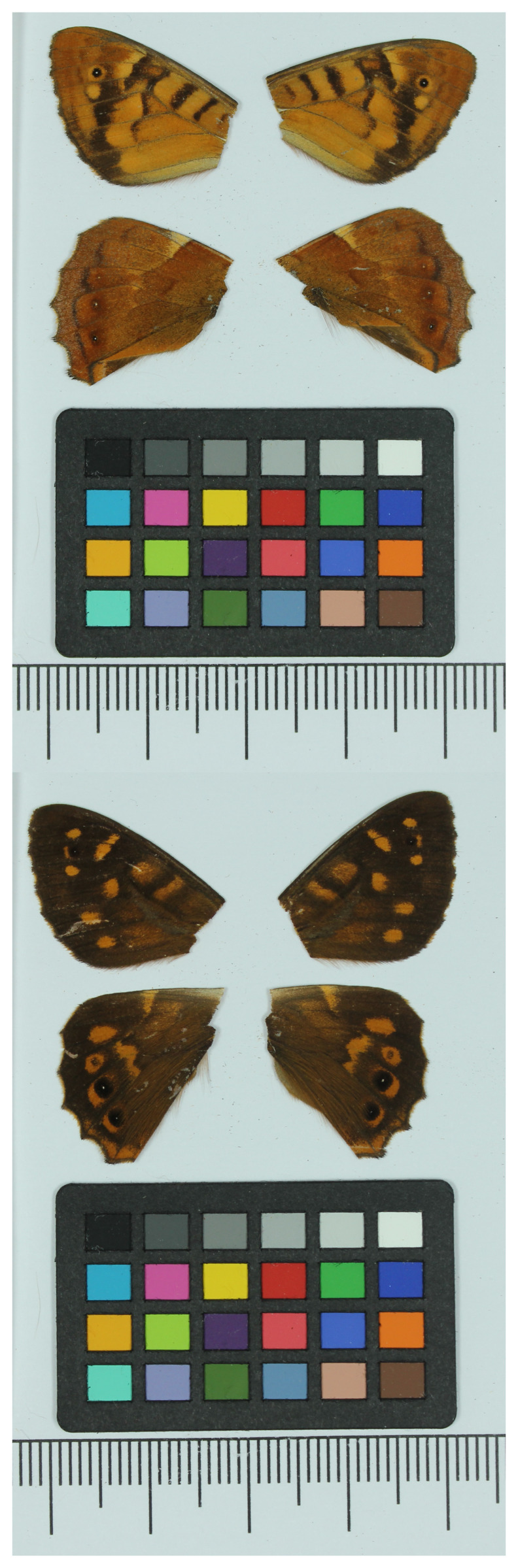
Voucher photograph of the *Pararge xiphia* (ilParXiph1) specimen used for genome sequencing.

We present a chromosome-level genome sequence of *Pararge xiphia*, generated as part of Project Psyche. The sequence data were derived from a male specimen ( Figure 1) collected in Madeira, Portugal.

## Methods

### Sample acquisition

The specimen used for genome sequencing was an adult male *Pararge xiphia* (specimen ID SAN28000346, ToLID ilParXiph1; Figure 1), collected from São Vicente, Madeira, Portugal (latitude 32.76, longitude –17.0167) on 2022-05-13. The specimen was collected by Roger Vila and Valéria Marques and identified by Roger Vila.

### Nucleic acid extraction

Protocols for high molecular weight (HMW) DNA extraction developed at the Wellcome Sanger Institute (WSI) Tree of Life Core Laboratory are available on protocols.io ( Howard *et al.*, 2025). The ilParXiph1 sample was weighed and triaged to determine the appropriate extraction protocol. Tissue from the whole organism was homogenised by powermashing using a PowerMasher II tissue disruptor.

HMW DNA was extracted in the WSI Scientific Operations core using the Automated MagAttract v2 protocol. DNA was sheared into an average fragment size of 12–20 kb following the Megaruptor®3 for LI PacBio protocol. Sheared DNA was purified by automated SPRI (solid-phase reversible immobilisation). The concentration of the sheared and purified DNA was assessed using a Nanodrop spectrophotometer and Qubit Fluorometer using the Qubit dsDNA High Sensitivity Assay kit. Fragment size distribution was evaluated by running the sample on the FemtoPulse system. For this sample, the final post-shearing DNA had a Qubit concentration of 66.75 ng/μL and a yield of 3 137.25 ng, with a fragment size of 15.1 kb.

### PacBio HiFi library preparation and sequencing

Library preparation and sequencing were performed at the WSI Scientific Operations core. Libraries were prepared using the SMRTbell Prep Kit 3.0 (Pacific Biosciences, California, USA), according to the manufacturer’s instructions. The kit includes reagents for end repair/A-tailing, adapter ligation, post-ligation SMRTbell bead clean-up, and nuclease treatment. Size selection and clean-up were performed using diluted AMPure PB beads (Pacific Biosciences). DNA concentration was quantified using a Qubit Fluorometer v4.0 (ThermoFisher Scientific) and the Qubit 1X dsDNA HS assay kit. Final library fragment size was assessed with the Agilent Femto Pulse Automated Pulsed Field CE Instrument (Agilent Technologies) using the gDNA 55 kb BAC analysis kit.

The sample was sequenced on a Revio instrument (Pacific Biosciences). The prepared library was normalised to 2 nM, and 15 μL was used for making complexes. Primers were annealed and polymerases bound to generate circularised complexes, following the manufacturer’s instructions. Complexes were purified using 1.2X SMRTbell beads, then diluted to the Revio loading concentration (200–300 pM) and spiked with a Revio sequencing internal control. The sample was sequenced on a Revio 25M SMRT cell. The SMRT Link software (Pacific Biosciences), a web-based workflow manager, was used to configure and monitor the run and to carry out primary and secondary data analysis.

Specimen details, sequencing platforms, and data yields are summarised in Table 1.

**Table 1.** Specimen and sequencing data for BioProject PRJEB81620.

Platform	PacBio HiFi	Hi-C
**ToLID**	ilParXiph1	ilParXiph1
**Specimen ID**	SAN28000346	SAN28000346
**BioSample (source individual)**	SAMEA115769909	SAMEA115769909
**BioSample (tissue)**	SAMEA115769929	SAMEA115769929
**Tissue**	whole organism	whole organism
**Instrument**	Revio	Illumina NovaSeq X
**Run accessions**	ERR13900452	ERR13907230
**Read count total**	2.04 million	600.67 million
**Base count total**	19.49 Gb	90.70 Gb

### Hi-C

***Sample preparation and crosslinking***

The Hi-C sample was prepared from 20–50 mg of frozen tissue from the whole organism of the ilParXiph1 sample using the Arima-HiC v2 kit (Arima Genomics). Following the manufacturer’s instructions, tissue was fixed and DNA crosslinked using TC buffer to a final formaldehyde concentration of 2%. The tissue was homogenised using the Diagnocine Power Masher-II. Crosslinked DNA was digested with a restriction enzyme master mix, biotinylated, and ligated. Clean-up was performed with SPRISelect beads before library preparation. DNA concentration was measured with the Qubit Fluorometer (Thermo Fisher Scientific) and Qubit HS Assay Kit. The biotinylation percentage was estimated using the Arima-HiC v2 QC beads.

***Hi-C library preparation and sequencing***

Biotinylated DNA constructs were fragmented using a Covaris E220 sonicator and size selected to 400–600 bp using SPRISelect beads. DNA was enriched with Arima-HiC v2 kit Enrichment beads. End repair, A-tailing, and adapter ligation were carried out with the NEBNext Ultra II DNA Library Prep Kit (New England Biolabs), following a modified protocol where library preparation occurs while DNA remains bound to the Enrichment beads. Library amplification was performed using KAPA HiFi HotStart mix and a custom Unique Dual Index (UDI) barcode set (Integrated DNA Technologies). Depending on sample concentration and biotinylation percentage determined at the crosslinking stage, libraries were amplified with 10–16 PCR cycles. Post-PCR clean-up was performed with SPRISelect beads. Libraries were quantified using the AccuClear Ultra High Sensitivity dsDNA Standards Assay Kit (Biotium) and a FLUOstar Omega plate reader (BMG Labtech).

Prior to sequencing, libraries were normalised to 10 ng/μL. Normalised libraries were quantified again to create equimolar and/or weighted 2.8 nM pools. Pool concentrations were checked using the Agilent 4200 TapeStation (Agilent) with High Sensitivity D500 reagents before sequencing. Sequencing was performed using paired-end 150 bp reads on the Illumina NovaSeq X.

Specimen details, sequencing platforms, and data yields are summarised in Table 1.

### Genome assembly

Prior to assembly of the PacBio HiFi reads, a database of *k*-mer counts ( *k* = 31) was generated from the filtered reads using FastK. GenomeScope2 ( Ranallo-Benavidez *et al.*, 2020) was used to analyse the *k*-mer frequency distributions, providing estimates of genome size, heterozygosity, and repeat content.

The HiFi reads were assembled using Hifiasm in Hi-C phasing mode ( Cheng *et al.*, 2021; Cheng *et al.*, 2022), producing two haplotypes. Hi-C reads ( Rao *et al.*, 2014) were mapped to the primary contigs using bwa-mem2 ( Vasimuddin *et al.*, 2019). Contigs were further scaffolded with Hi-C data in YaHS ( Zhou *et al.*, 2023), using the --break option for handling potential misassemblies. The scaffolded assemblies were evaluated using Gfastats ( Formenti *et al.*, 2022), BUSCO ( Manni *et al.*, 2021) and MERQURY.FK ( Rhie *et al.*, 2020).

The mitochondrial genome was assembled using MitoHiFi ( Uliano-Silva *et al.*, 2023), which runs MitoFinder ( Allio *et al.*, 2020) and uses these annotations to select the final mitochondrial contig and to ensure the general quality of the sequence.

### Assembly curation

The assembly was decontaminated using the Assembly Screen for Cobionts and Contaminants ( ASCC) pipeline. TreeVal was used to generate the flat files and maps for use in curation. Manual curation was conducted primarily in PretextView and HiGlass ( Kerpedjiev *et al.*, 2018). Scaffolds were visually inspected and corrected as described by Howe *et al*. (2021). Manual corrections included 22 breaks and 76 joins. This reduced the scaffold count by 31.9% and increased the scaffold N50 by 3.1%. The curation process is described at https://gitlab.com/wtsi-grit/rapid-curation. PretextSnapshot was used to generate a Hi-C contact map of the final assembly.

### Assembly quality assessment

The Merqury.FK tool ( Rhie *et al.*, 2020), run in a Singularity container ( Kurtzer *et al.*, 2017), was used to evaluate *k*-mer completeness and assembly quality for both haplotypes using the *k*-mer database ( *k* = 31) computed prior to genome assembly. The analysis outputs included assembly QV scores and completeness statistics.

The genome was analysed using the BlobToolKit pipeline, a Nextflow ( Di Tommaso *et al.*, 2017) implementation of the earlier Snakemake version ( Challis *et al.*, 2020). The pipeline aligns PacBio reads using minimap2 ( Li, 2018) and SAMtools ( Danecek *et al.*, 2021) to generate coverage tracks. It runs BUSCO ( Manni *et al.*, 2021) using lineages identified from the NCBI Taxonomy ( Schoch *et al.*, 2020). For the three domain-level lineages, BUSCO genes are aligned to the UniProt Reference Proteomes database ( Bateman *et al.*, 2023) using DIAMOND blastp ( Buchfink *et al.*, 2021). The genome is divided into chunks based on the density of BUSCO genes from the closest taxonomic lineage, and each chunk is aligned to the UniProt Reference Proteomes database with DIAMOND blastx. Sequences without hits are chunked using seqtk and aligned to the NT database with blastn ( Altschul *et al.*, 1990). The BlobToolKit suite consolidates all outputs into a blobdir for visualisation. The BlobToolKit pipeline was developed using nf-core tooling ( Ewels *et al.*, 2020) and MultiQC ( Ewels *et al.*, 2016), with containerisation through Docker ( Merkel, 2014) and Singularity ( Kurtzer *et al.*, 2017).

We used lep_busco_painter to paint Merian elements along chromosomes ( Wright *et al.*, 2024). Merian elements represent the 32 ancestral linkage groups in Lepidoptera. The painting process utilised BUSCO gene locations from the lepidoptera_odb10 set ( Kriventseva *et al.*, 2019) and chromosome lengths from NCBI Datasets. Each complete BUSCO gene (both single-copy and duplicated) was assigned to a Merian element based on a reference database, then plotted along chromosomes drawn to scale.

## Genome sequence report

### Sequence data

**Figure 2.**
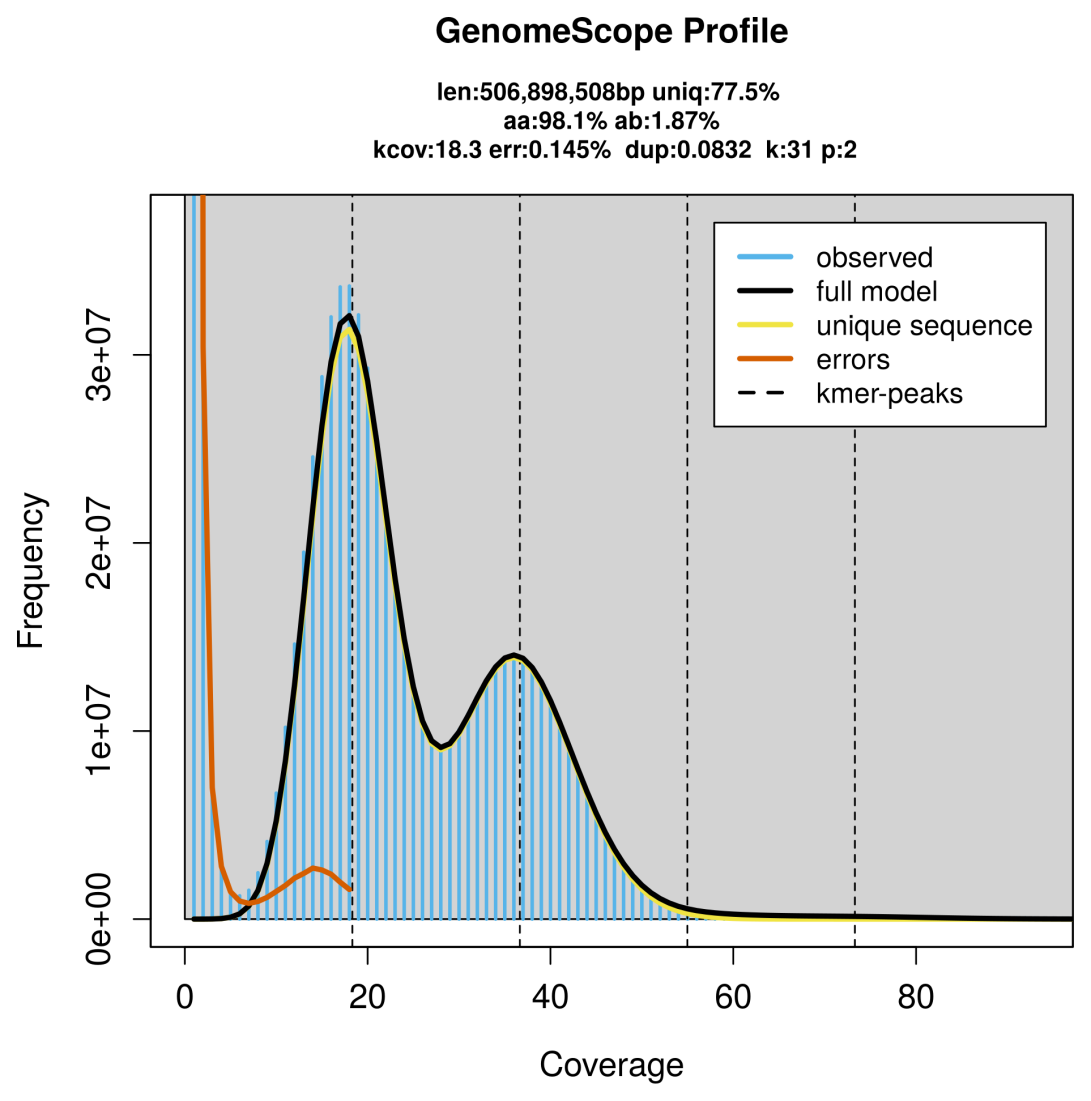
Frequency distribution of *k*-mers generated using GenomeScope2. The plot shows observed and modelled *k*-mer spectra, providing estimates of genome size, heterozygosity, and repeat content based on unassembled sequencing reads.

PacBio sequencing of the *Pararge xiphia* specimen generated 19.49 Gb (gigabases) from 2.04 million reads, which were used to assemble the genome. GenomeScope2.0 analysis estimated the haploid genome size at 507.17 Mb, with a heterozygosity of 1.87% and repeat content of 22.52% ( Figure 2). These estimates guided expectations for the assembly. Based on the estimated genome size, the sequencing data provided approximately 37× coverage. Hi-C sequencing produced 90.70 Gb from 600.67 million reads, which were used to scaffold the assembly. Table 1 summarises the specimen and sequencing details.

### Assembly statistics

The genome was assembled into two haplotypes using Hi-C phasing. Haplotype 1 was curated to chromosome level, while haplotype 2 was assembled to scaffold level. The final assembly has a total length of 528.41 Mb in 91 scaffolds, with 143 gaps, and a scaffold N50 of 20.0 Mb ( Table 2).

**Table 2.** Genome assembly statistics.

**Assembly name**	ilParXiph1.hap1.1	ilParXiph1.hap2.1
**Assembly accession**	GCA_964330365.1	GCA_964330265.1
**Assembly level**	chromosome	scaffold
**Span (Mb)**	528.41	523.51
**Number of chromosomes**	28	scaffold-level
**Number of contigs**	234	221
**Contig N50**	5.81 Mb	5.95 Mb
**Number of scaffolds**	91	78
**Scaffold N50**	20.0 Mb	19.76 Mb
**Longest scaffold length (Mb)**	29.42	-
**Sex chromosomes**	Z	-
**Organelles**	Mitochondrion: 15.27 kb	-

**Figure 3.**
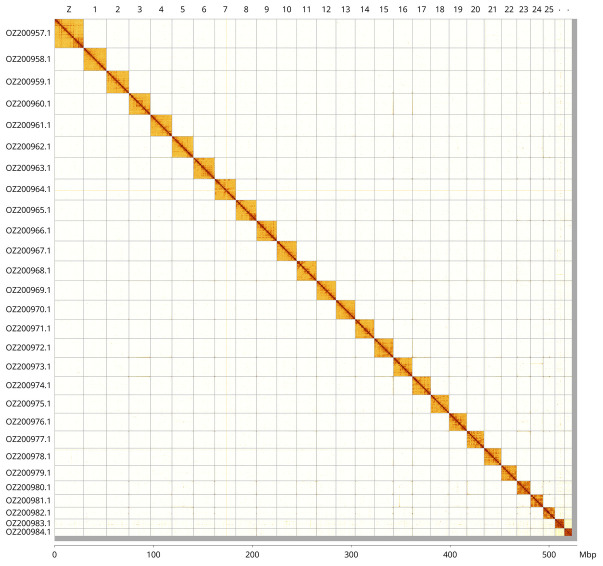
Hi-C contact map of the *Pararge xiphia* genome assembly. Assembled chromosomes are shown in order of size and labelled along the axes, with a megabase scale shown below. The plot was generated using PretextSnapshot.

**Figure 4.**
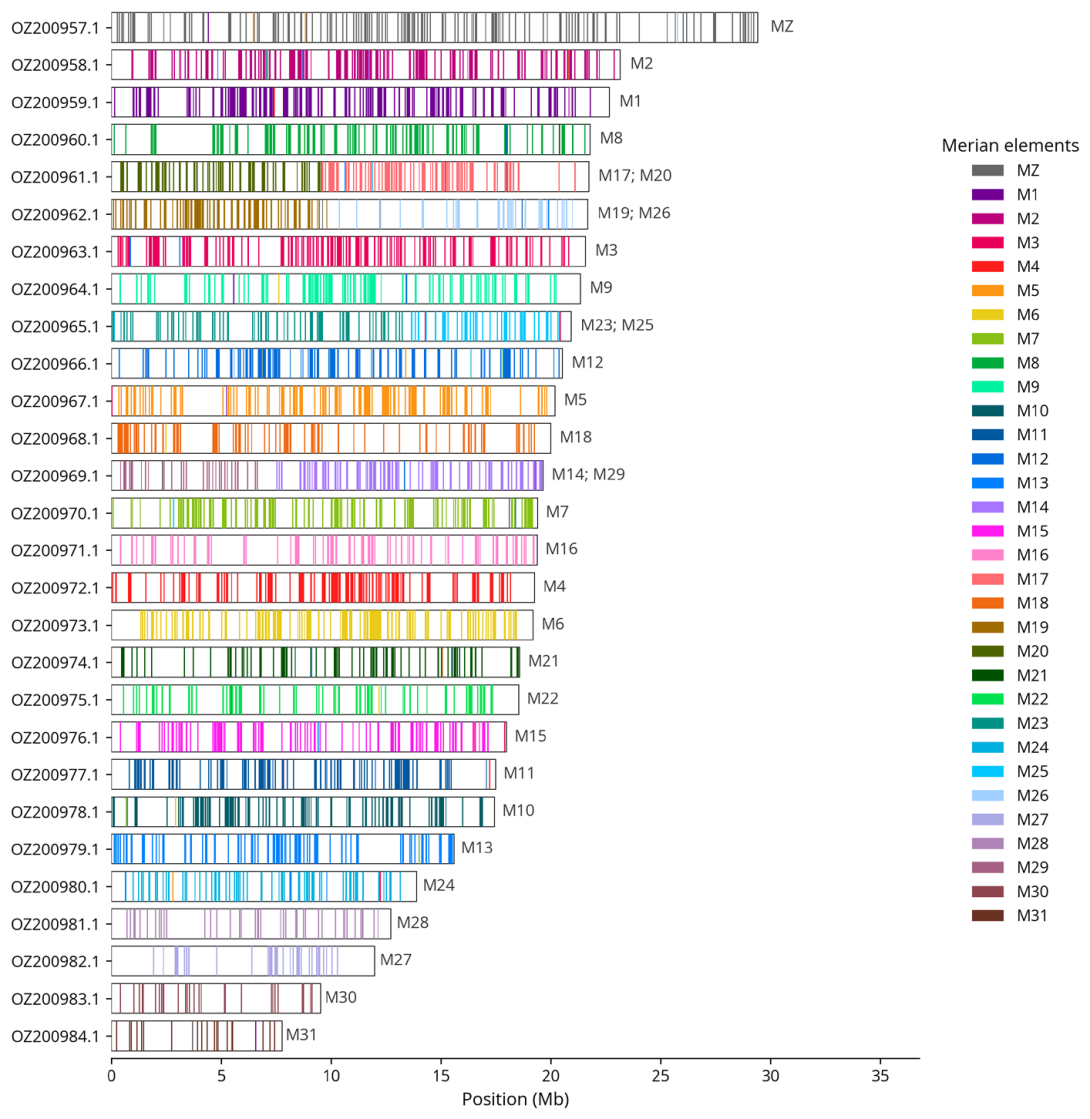
Merian elements painted across chromosomes in the ilParXiph1.hap1.1 assembly of *Pararge xiphia*. Chromosomes are drawn to scale, with the positions of orthologues shown as coloured bars. Each orthologue is coloured by the Merian element that it belongs to. All orthologues which could be assigned to Merian elements are shown.

Most of the assembly sequence (99.02%) was assigned to 28 chromosomal-level scaffolds, representing 27 autosomes and the Z sex chromosome. These chromosome-level scaffolds, confirmed by Hi-C data, are named according to size ( Figure 3; Table 3). Chromosome painting with Merian elements illustrates the distribution of orthologues along chromosomes and highlights patterns of chromosomal evolution relative to Lepidopteran ancestral linkage groups ( Figure 4). Chromosome Z was identified through the detection of ancestral BUSCO genes. The exact order and orientation of the contigs on chromosome 26 (5,400 - 5,500 Kbp) are unknown.

**Table 3.** Chromosomal pseudomolecules in the haplotype 1 genome assembly of *Pararge xiphia* ilParXiph1.

INSDC accession	Molecule	Length (Mb)	GC%	Assigned Merian elements
OZ200958.1	1	23.15	36.50	M2
OZ200959.1	2	22.67	37	M1
OZ200960.1	3	21.79	37	M8
OZ200961.1	4	21.72	37	M17;M20
OZ200962.1	5	21.67	37	M19;M26
OZ200963.1	6	21.57	37	M3
OZ200964.1	7	21.34	36.50	M9
OZ200965.1	8	20.92	37	M23;M25
OZ200966.1	9	20.52	37	M12
OZ200967.1	10	20.19	37	M5
OZ200968.1	11	20	36.50	M18
OZ200969.1	12	19.64	37	M14;M29
OZ200970.1	13	19.39	37	M7
OZ200971.1	14	19.37	37	M16
OZ200972.1	15	19.26	37	M4
OZ200973.1	16	19.18	37	M6
OZ200974.1	17	18.58	37	M21
OZ200975.1	18	18.54	37	M22
OZ200976.1	19	17.98	37.50	M15
OZ200977.1	20	17.48	37.50	M11
OZ200978.1	21	17.43	37.50	M10
OZ200979.1	22	15.60	37.50	M13
OZ200980.1	23	13.88	38.50	M24
OZ200981.1	24	12.72	38	M28
OZ200982.1	25	11.97	38.50	M27
OZ200983.1	26	9.51	40	M30
OZ200984.1	27	7.77	39	M31
OZ200957.1	Z	29.42	37	MZ

The mitochondrial genome was also assembled (length 15.27 kb, OZ200985.1). This sequence is included as a contig in the multifasta file of the genome submission and as a standalone record.

### Assembly quality metrics

**Figure 5.**
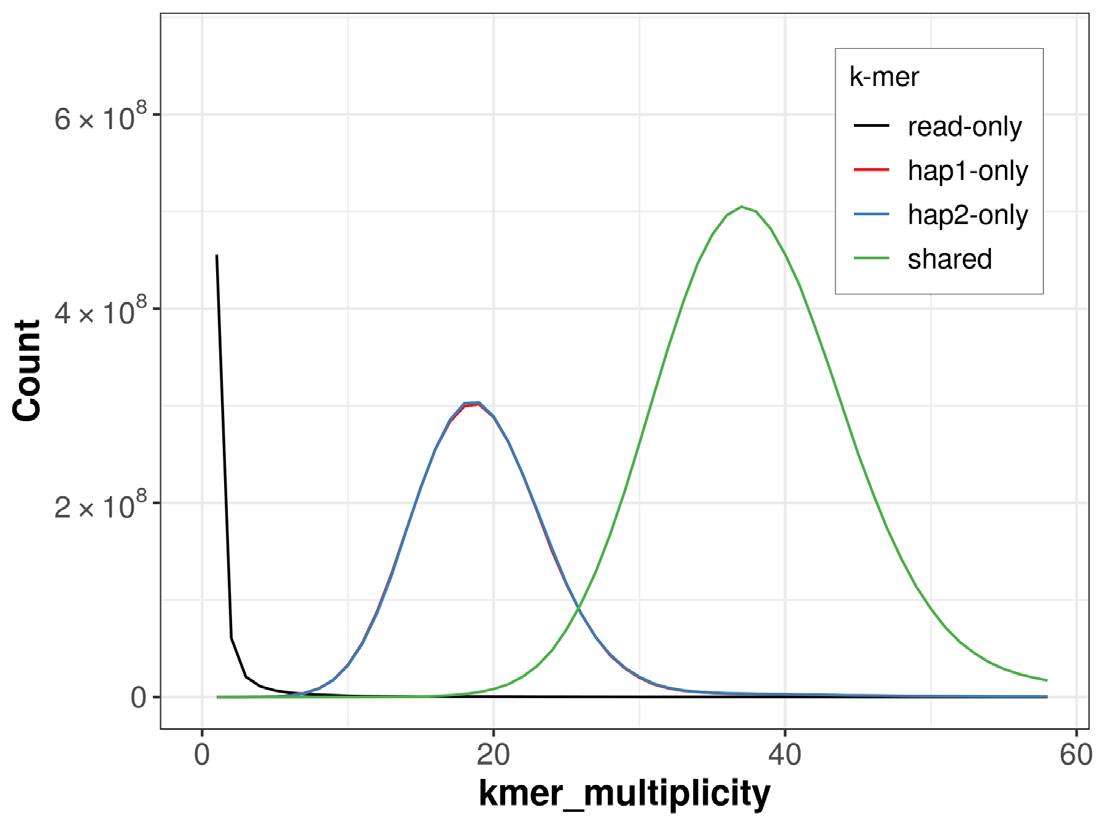
Evaluation of *k*-mer completeness using MerquryFK. This plot illustrates the recovery of *k*‐mers from the original read data in the final assemblies. The horizontal axis represents *k*‐mer multiplicity, and the vertical axis shows the number of *k*‐mers. The black curve represents *k*‐mers that appear in the reads but are not assembled. The green curve (the homozygous peak) corresponds to *k*‐mers shared by both haplotypes and the red and blue curves (the heterozygous peaks) show *k*‐mers found only in one of the haplotypes.

**Figure 6.**
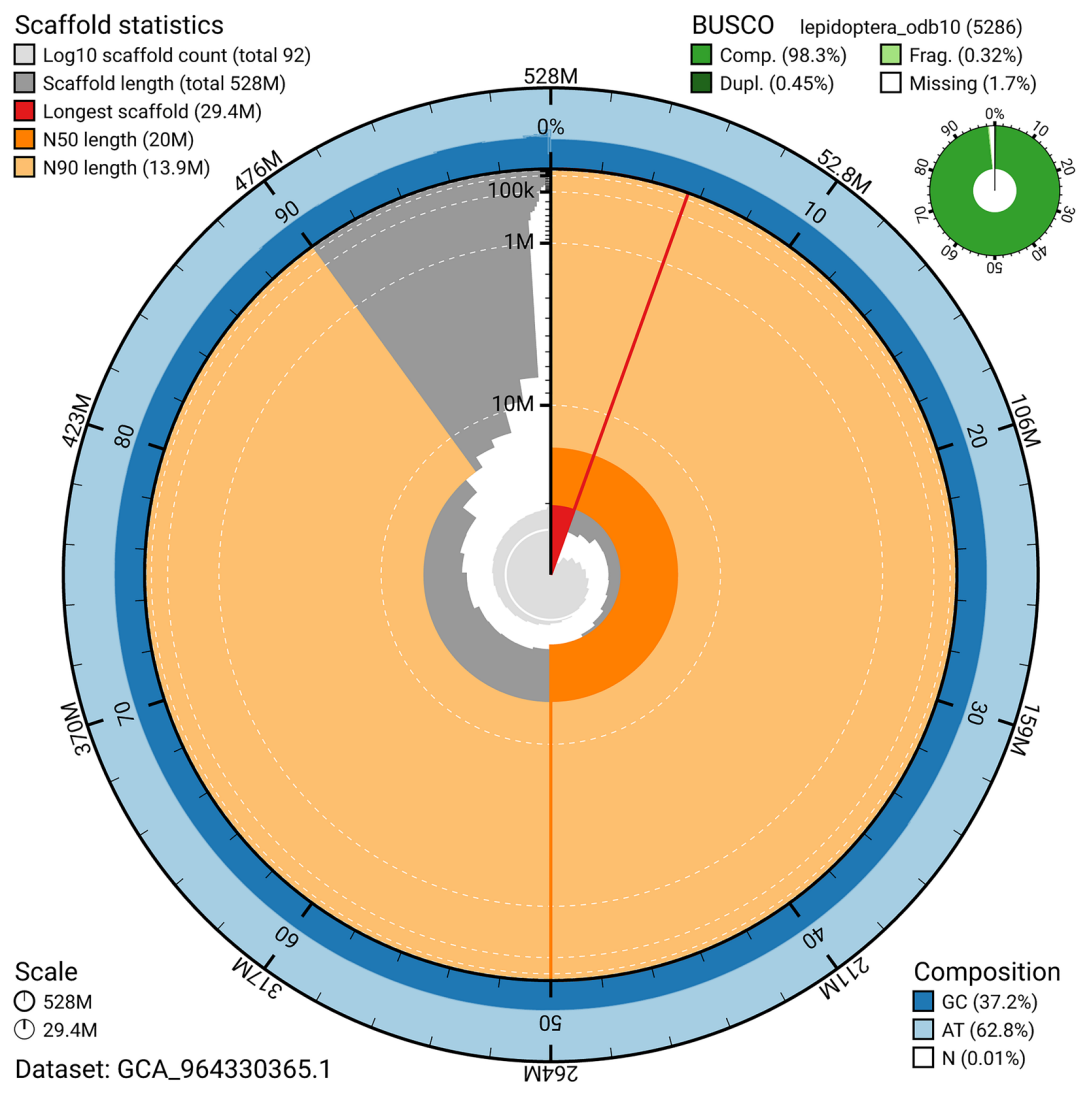
Assembly metrics for ilParXiph1.hap1.1. The BlobToolKit snail plot provides an overview of assembly metrics and BUSCO gene completeness. The circumference represents the length of the whole genome sequence, and the main plot is divided into 1,000 bins around the circumference. The outermost blue tracks display the distribution of GC, AT, and N percentages across the bins. Scaffolds are arranged clockwise from longest to shortest and are depicted in dark grey. The longest scaffold is indicated by the red arc, and the deeper orange and pale orange arcs represent the N50 and N90 lengths. A light grey spiral at the centre shows the cumulative scaffold count on a logarithmic scale. A summary of complete, fragmented, duplicated, and missing BUSCO genes in the set is presented at the top right. An interactive version of this figure can be accessed on the BlobToolKit viewer.

**Figure 7.**
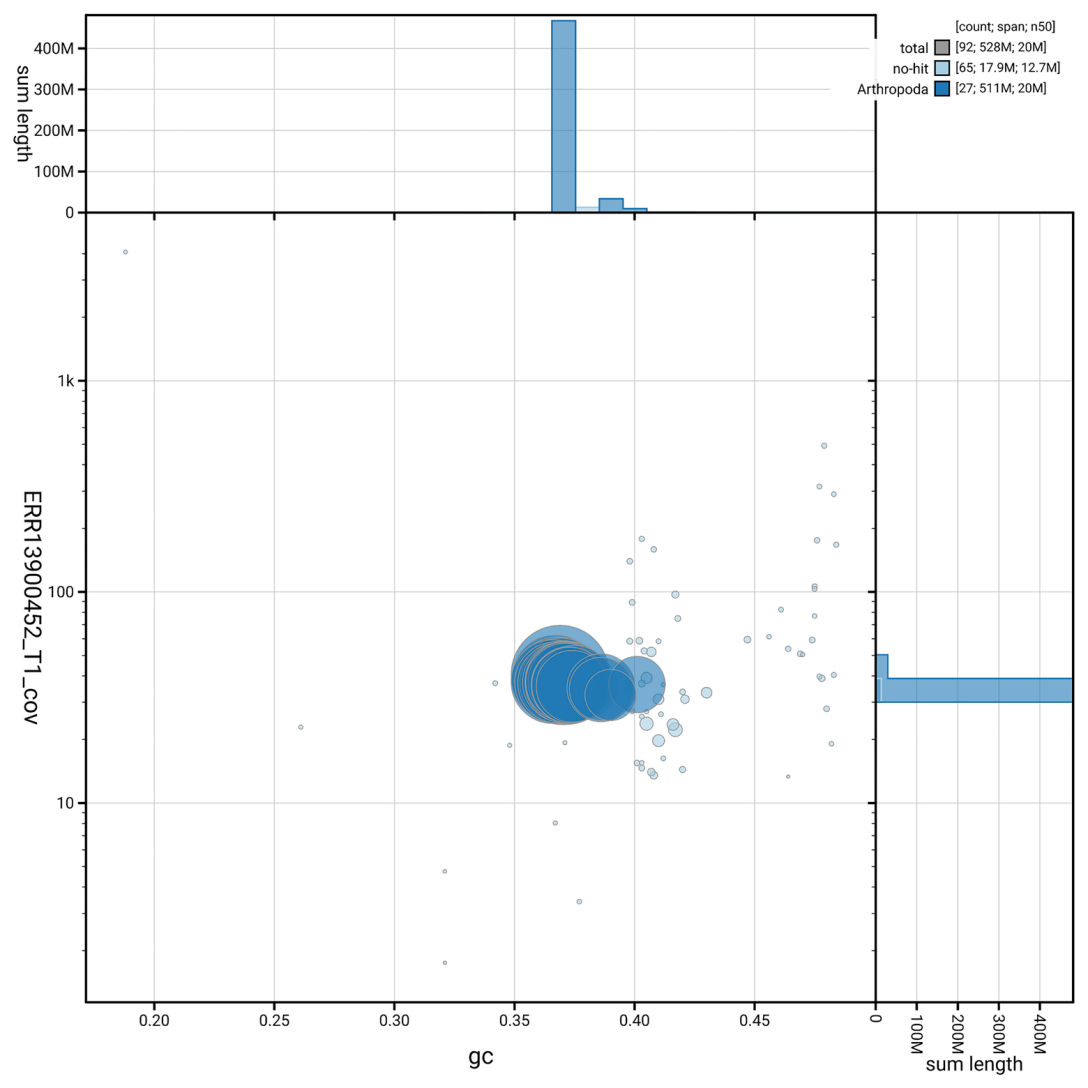
BlobToolKit GC-coverage plot for ilParXiph1.hap1.1. Blob plot showing sequence coverage (vertical axis) and GC content (horizontal axis). The circles represent scaffolds, with the size proportional to scaffold length and the colour representing phylum membership. The histograms along the axes display the total length of sequences distributed across different levels of coverage and GC content. An interactive version of this figure is available on the BlobToolKit viewer.

For haplotype 1, the estimated QV is 65.9, and for haplotype 2, 65.5. When the two haplotypes are combined, the assembly achieves an estimated QV of 65.7. The *k*-mer completeness is 68.88% for haplotype 1, 68.92% for haplotype 2, and 99.71% for the combined haplotypes ( Figure 5). BUSCO analysis using the lepidoptera_odb10 reference set ( *n* = 5 286) identified 98.3% of the expected gene set (single = 97.8%, duplicated = 0.5%) in haplotype 1. For haplotype 2, BUSCO analysis identified 98.4% of the expected gene set (single = 98.0%, duplicated = 0.4%). The snail plot in Figure 6 summarises the scaffold length distribution and other assembly statistics for haplotype 1. The blob plot in Figure 7 shows the distribution of scaffolds by GC proportion and coverage for haplotype 1.

Table 4 lists the assembly metric benchmarks adapted from Rhie *et al.* (2021) the Earth BioGenome Project Report on Assembly Standards September 2024. The EBP metric, calculated for the haplotype 1, is **6.C.Q65**, meeting the recommended reference standard.

**Table 4.** Earth Biogenome Project summary metrics for the *Pararge xiphia* assembly.

Measure	Value	Benchmark
EBP summary (haplotype 1)	6.C.Q65	6.C.Q40
Contig N50 length	5.81 Mb	≥ 1 Mb
Scaffold N50 length	20 Mb	= chromosome N50
Consensus quality (QV)	Haplotype 1: 65.9; haplotype 2: 65.5; combined: 65.7	≥ 40
*k*-mer completeness	Haplotype 1: 68.88%; Haplotype 2: 68.92%; combined: 99.71%	≥ 95%
BUSCO	C:98.3% [S:97.8%; D:0.5%]; F:0.3%; M:1.4%; n:5 286	S > 90%; D < 5%
Percentage of assembly assigned to chromosomes	99.02%	≥ 90%

**Notes:** EBP summary uses log10(Contig N50); chromosome-level (C) or log10(Scaffold N50); Q (Merqury QV). BUSCO: C=complete; S=single-copy; D=duplicated; F=fragmented; M=missing; n=orthologues

### Wellcome Sanger Institute – Legal and Governance

The materials that have contributed to this genome note have been supplied by a Tree of Life collaborator. The Wellcome Sanger Institute employs a process whereby due diligence is carried out proportionate to the nature of the materials themselves, and the circumstances under which they have been/are to be collected and provided for use. The purpose of this is to address and mitigate any potential legal and/or ethical implications of receipt and use of the materials as part of the research project, and to ensure that in doing so, we align with best practice wherever possible. The overarching areas of consideration are:

- Ethical review of provenance and sourcing of the material
- Legality of collection, transfer and use (national and international).

Each transfer of samples is undertaken according to a Research Collaboration Agreement or Material Transfer Agreement entered into by the Tree of Life collaborator, Genome Research Limited (operating as the Wellcome Sanger Institute), and in some circumstances, other Tree of Life collaborators.

## Data Availability

European Nucleotide Archive: Pararge xiphia (Madeiran speckled wood butterfly). Accession number PRJEB81620. The genome sequence is released openly for reuse. The *Pararge xiphia* genome sequencing initiative is part of the Sanger Institute Tree of Life Programme (PRJEB43745) and Project Psyche (PRJEB71705). All raw sequence data and the assembly have been deposited in INSDC databases. The genome will be annotated using available RNA-Seq data and presented through Ensembl at the European Bioinformatics Institute. Raw data and assembly accession identifiers are reported in Table 1 and Table 2. Pipelines used for genome assembly at the WSI Tree of Life are available at https://pipelines.tol.sanger.ac.uk/pipelines. Table 5 lists software versions used in this study.

## Author information

Contributors are listed at the following links:

- Members of the Wellcome Sanger Institute Tree of Life Management, Samples and Laboratory team
- Members of Wellcome Sanger Institute Scientific Operations – Sequencing Operations
- Members of the Wellcome Sanger Institute Tree of Life Core Informatics team
- Members of the Tree of Life Core Informatics collective
- Members of the Project Psyche Community
