# Supplementary material for: The genome sequence of the Madeiran Speckled Wood, Pararge xiphia Hübner, 1819 (Lepidoptera: Nymphalidae)
Source: Wellcome Open Res. 2026 Feb 9;11:94. [Version 1] doi: 10.12688/wellcomeopenres.25814.1 (PMC13022564; doi:10.12688/wellcomeopenres.25814.1)
Supplement: Peer review history

# Reviewer report: Reviewer response for version 1

**DOI:** 10.21956/wellcomeopenres.28415.r150172

**Published:** 2026-03-26

**Authors:** Yoshiaki Morino

**Affiliations:** 1 University of Tsukuba, Tsukuba, Japan

This manuscript presents a high-quality reference genome assembly for *Pararge xiphia* generated from a male specimen collected in Madeira, using PacBio HiFi and Hi-C data. The assembly is reported as a phased diploid resource comprising two haplotypes of 528.41 Mb and 523.51 Mb, respectively. For haplotype 1, 99.02% of the assembly is assigned to 28 chromosomal pseudomolecules, including the Z chromosome. The contiguity statistics are also strong, with a contig N50 of 5.81 Mb and a scaffold N50 of 20.0 Mb for haplotype 1, and the mitochondrial genome has additionally been assembled as a 15.27 kb sequence. Assembly quality is high, with an estimated QV of 65.9 for haplotype 1 and BUSCO completeness of 98.3% against the lepidoptera_odb10 dataset (97.8% single-copy, 0.5% duplicated). 

 The manuscript also gives clear and thorough documentation of sample processing, sequencing, assembly, curation, and data release, which makes the study transparent and reproducible. It also includes an informative overview of the biological background of the species, helping to place the genome resource in a broader evolutionary and taxonomic context. Overall, this appears to be a valuable genome resource that should support future work on butterfly genomics, comparative biology, and evolutionary studies.

Are sufficient details of methods and materials provided to allow replication by others?

Yes

Is the rationale for creating the dataset(s) clearly described?

Yes

Are the datasets clearly presented in a useable and accessible format?

Yes

Are the protocols appropriate and is the work technically sound?

Yes

Reviewer Expertise:

Evolutionary developmental biology, Invertebrate

I confirm that I have read this submission and believe that I have an appropriate level of expertise to confirm that it is of an acceptable scientific standard.

# Reviewer report: Reviewer response for version 1

**DOI:** 10.21956/wellcomeopenres.28415.r148660

**Published:** 2026-03-12

**Authors:** Wai Lok So

**Affiliations:** 1 The Hang Seng University of Hong Kong, Hong Kong, Hong Kong

The manuscript presents a genome assembly of *Pararge xiphia* as a part of the Project Psyche initiative. The sequencing and assembly methods are technically sound, yielding an excellent scaffolding of haplotype 1, including the z sex chromosome and a complete mitochondrial genome. The generated resources are transparent and accessible. The resulting resource would be very useful for research on Lepidoptera evolution, conservation and genomics.

 I have no major concerns with the manuscript, except for the following minor comments:

 1. Was the butterfly identified morphologically or by PCR of certain marker genes?

 2. There are two specimen photos shown in Figure 1 with the same ID. Are they the front and back side of the same butterfly? Is so, please kindly state clearly in the figure legend to avoid misunderstanding.

Are sufficient details of methods and materials provided to allow replication by others?

Yes

Is the rationale for creating the dataset(s) clearly described?

Yes

Are the datasets clearly presented in a useable and accessible format?

Yes

Are the protocols appropriate and is the work technically sound?

Yes

Reviewer Expertise:

evolution, genomics, arthropods

I confirm that I have read this submission and believe that I have an appropriate level of expertise to confirm that it is of an acceptable scientific standard.
